# Production, Bioprocessing and Anti-Proliferative Activity of Camptothecin from *Penicillium chrysogenum*, “An Endozoic of Marine Sponge, *Cliona* sp.”, as a Metabolically Stable Camptothecin Producing Isolate

**DOI:** 10.3390/molecules27093033

**Published:** 2022-05-09

**Authors:** Ashraf S. A. El-Sayed, Wafaa H. B. Hassan, Sherouk Hussein Sweilam, Mohammed Hamed Saeed Alqarni, Zeinab I. El Sayed, Mahmoud M. Abdel-Aal, Eman Abdelsalam, Sahar Abdelaziz

**Affiliations:** 1 Enzymology and Fungal Biotechnology Lab, Botany and Microbiology Department, Faculty of Science, Zagazig University, Zagazig 44519, Egypt; 2 Department of Pharmacognosy, Faculty of Pharmacy, Zagazig University, Zagazig 44519, Egypt; wafaahbh@zu.edu.eg (W.H.B.H.); zeinab@gmail.com (Z.I.E.S.); mahmoud.ibrahim@su.edu.eg (M.M.A.-A.); gameela_86@yahoo.com (E.A.); sah_abdelaziz@zu.edu.eg (S.A.); 3 Department of Pharmacognosy, Faculty of Pharmacy, Prince Sattam Bin Abdulaziz University, Al-Kharj 11942, Saudi Arabia; s.sweilam@psau.edu.sa (S.H.S.); m.alqarni@psau.edu.sa (M.H.S.A.); 4 Department of Pharmacognosy, Faculty of Pharmacy, Egyptian Russian University, Cairo-Suez Road, Badr City 11829, Egypt

**Keywords:** camptothecin, *Penicillium chrysogenum*, *Cliona* sp., anticancer activity, LC-MS/MS

## Abstract

Exploring the metabolic potency of fungi as camptothecin producers raises the hope of their usage as an industrial source of camptothecin, due to their short-life span and the feasibility of metabolic engineering. However, the tiny yield and loss of camptothecin productivity of fungi during storage and sub-culturing are challenges that counteract this approach. Marine fungi could be a novel source for camptothecin production, with higher yield and reliable metabolic sustainability. The marine fungal isolate *Penicillium chrysogenum* EFBL # OL597937.1 derived from the sponge “*Cliona* sp.” has been morphologically identified and molecularly confirmed, based on the Internal Transcribed Spacer sequence, exhibiting the highest yield of camptothecin (110 μg/L). The molecular structure and chemical identity of *P. chrysogenum* derived camptothecin has been resolved by HPLC, FTIR and LC-MS/MS analyses, giving the same spectroscopic profiles and mass fragmentation patterns as authentic camptothecin. The extracted camptothecin displayed a strong anti-proliferative activity towards HEP-2 and HCT-116 (IC_50_ values 0.33–0.35 µM). The yield of camptothecin was maximized by nutritional optimization of *P. chrysogenum* with a Plackett-Burman design, and the productivity of camptothecin increased by 1.8 fold (200 µg/L), compared to control fungal cultures. Upon storage at 4 °C as slope culture for 8 months, the productivity of camptothecin for *P. chrysogenum* was reduced by 40% compared to the initial culture. Visual fading of the mycelial pigmentation of *P. chrysogenum* was observed during fungal storage, matched with loss of camptothecin productivity. Methylene chloride extracts of *Cliona* sp. had the potency to completely restore the camptothecin productivity of *P. chrysogenum*, ensuring the partial dependence of the expression of the camptothecin biosynthetic machinery of *P. chrysogenum* on the chemical signals derived from the sponge, or the associated microbial flora. This is the first report describing the feasibility of *P. chrysogenum*, endozoic of *Cliona* sp., for camptothecin production, along with reliable metabolic biosynthetic stability, which could be a new platform for scaling-up camptothecin production.

## 1. Introduction

Cancer is the major cause of death worldwide, with an annual increase in the number of cases. With these elevated mortality rates, exploring novel approaches to cancer therapy is indispensible. Camptothecin was first isolated from *Camptotheca acuminata* in China [1]. Water soluble camptothecin derivatives, Topotecan and Irinotecan, have been approved by the Food and Drug Administration (FDA) as a universal drug for ovarian, cell lung cancer, colorectal carcinoma refractory and other metastatic colorectal cancers [2,3,4,5]. The anticancer activity of Camptothecin stems from its higher affinity and interaction with Topoisomerase-I (Topo I), an enzyme that regulates the DNA topology during replication, recombination and transcription. The topoisomerase I is usually involved in relaxation of DNA supercoiling by creating a nick in the single strand of DNA to release the supercoils generated from the multiple replication of tumor cells, making an ester linkage with the 3′ end of nicked DNA through its catalytic tyrosine [6,7,8]. Inhibition of DNA Topo I by camptothecin causes a protein-DNA breakage in various types of tumor cells [9,10]. Camptothecin (C_20_H_16_N_2_O_4_) has five cyclic structural rings, three rings of pyrrolo-(3,4-β)-quinoline (A, B, and C), coupled with a pyridone (ring D) at position 20, and one chiral center within the α- hydroxy lactone ring with (S) configuration (E ring).

Commercially, camptothecin is the third largest commercial anticancer drug after Taxol and vincristine [8]. However, there are challenges that impede the clinical applications of this compound. (1) Poor water solubility with severe gastrointestinal toxicity of the core camptothecin compound [11,12] is a limitation which has been resolved by developing highly water soluble camptothecin derivatives 10-hydroxycamptothecin, topotecan and irinotecan [5,13]. (2) The tiny yield of camptothecin core from its natural source “*C. acuminata*” has resulted in a destructive harvesting of this plant in China and India to fulfill the heavy demand that exerts a negative impact on the natural ecosystem [14,15,16]. Moreover, low abundance, extraction difficulties, steric complexity, and bulky compounds are the major hurdles that limit dependence on this plant as a natural source [17,18]. Thus, searching for alternative approaches with higher camptothecin productivity is the current challenge. Fungal endophytes inhabiting camptothecin-producing plants have been reported as a powerful source for camptothecin production, as reviewed by [19,20,21,22]. *Entrophospora infrequens*, endophyte of *Nothapodytes foetida*. [23,24], has been reported as the first fungal endophyte producing camptothecin. Consequently, a plethora of reports recording the potency of fungal endophytes as camptothecin producers, for example *Fusarium solani*, endophyte of *Apodytes dimidiate* [25,26], *Trichoderma atroviride*, endophytes of *C. acuminata* [20], *Alternaria alternate*, *Fomitopsis* sp., *Phomopsis* sp., endophytes of *Miquelia dentata*, have been reported as camptothecin and camptothecin-derivative producers [27]. The metabolic potency of fungi for camptothecin production raises hope for large-scale production of this compound due to the fast growth rate of fungi, accessibility of bulk biomass production, independence from environmental and ecological fluctuations, and the feasibility of metabolic manipulation of fungi [2,22,28]. Nevertheless, the tiny yield and the loss of camptothecin productivity during storage and subculturing are the main limiting challenges that prevent the further implementation of fungi for industrial trials [29,30,31,32,33]. Screening for novel fungal isolates from new biological entities is one of the most promising and reliable sources for fungi with diverse prospective metabolic identities. The fungal isolates inhabiting organisms that normally live under abnormal conditions could be an untapped reservoir of unique biologically active compounds with diverse chemical structures.

Recently, marine fungi have been reported as an untapped repertoire for novel bioactive secondary metabolites, with diverse biological and pharmaceutical activities [34]. However, the biological and metabolic identities of these marine fungi have received less attention and less exploration, compared to their terrestrial counterparts [35]. Marine fungi could be a potential candidate for the discovery of novel compounds with unique chemical skeletons and scaffolds that can be modified to produce novel bioactive and pharmaceutical activity [36,37]. Thus, isolation of camptothecin-producing fungi with a prospective, sustainable and stable biosynthetic machinery for the derivation of camptothecin from the marine sponge is the objective of this study.

## 2. Materials and Methods

### 2.1. Collection of Marine Sponges, Isolation and Morphological Identification of the Endozoic Fungi

Two marine sponges, *Cliona* sp. and *Hymedesmia* sp. belonging to class *Demospongiae*, were collected from the Red Sea, 20 km away from Sharm El sheikh, Egypt [27°45′57.8″ N 34°22′10.8″ E] using scuba diving at a depth of 8:10 m off, during Nov-Dec /2018. The collected sponges were immediately frozen and kept at −20 °C to maintain the normal endogenous microbial flora. The sponges were identified by a staff member of the Marine Science Department, Faculty of Science, Suez Canal University. The sponges were brought to the laboratory in a sterile ice box, washed thoroughly with sterile sea water to eliminate adherent surface debris prior to isolation of their associated fungi, and sectioned into small segments of approximately 1 cm × 1 cm. The parts were surface sterilized with EtOH 70% (vol/vol) for 1 min, followed by 2.5% sodium hypochlorite for 2 min, then rinsed with sterile sea water to avoid epiphytic microbes [38,39,40,41]. The surface sterilized sponge parts were placed on the surface of potato dextrose agar (200 g potato extract, 20 g glucose, 20 g agar per liter), malt yeast agar (MYA), malt extract agar (MA) and Czapek’s-Dox media (3.0 g NaNO_3_, 1.0 g KH_2_PO_4_, 0.5 g MgSO_4_·7H_2_O, 0.5 g KCl, 0.01 g FeSO_4_.7H_2_O, 30 g glucose, 20 g agar) dissolved in 1 L of distilled water. Chloramphenicol (0.2 g) was added to the media and the plates with different media and sponge parts were incubated for 15 days at 30 °C [42,43,44]. The developed fungal colonies were purified by subculturing on the same media, and the purified fungal isolates were kept as slope cultures at 4 °C.

The recovered fungal isolates were morphologically identified by central inoculation to the plates of potato dextrose agar (BD, Difco, Cat# DF0549-17-9), Czapek’s-Dox agar [42,43,44], and incubated for 10 days at 30 °C, and the developed colonies were examined daily, based on their macromorphological and micromorphological features, to species level according to the universal keys [45,46,47].

### 2.2. Screening and Chromatographic Analyses of Camptothecin from the Potent Fungi

The recovered endophytic fungi from the marine sponges were screened for camptothecin production by growing in potato dextrose broth (PDB) (BD Difco, Cat# DF0549-17-9) [48]. Each fungal isolate (2 agar plugs of 5 mm) of 6 days old PDA culture was inoculated into 50 mL PDB medium/250 mL Erlenmeyer flask. Three biological replicates of each fungal isolate were conducted. After incubation of the cultures at 30 °C for 10 days, the cultures were filtered by sterile cheesecloth, and the filtrates were centrifuged at 5000 rpm to remove any particulates. Camptothecin was extracted from the supernatant by CHCl_3_:MeOH (4:1), the organic phase was concentrated to a crude oily extract. The extract was fractionated on TLC (Merck 1 mm (20 cm × 20 cm), Silica gel 60 F254, Merck KGaA, Darmstadt, Germany) with the developing solvent system chloroform: methanol (9:1, *v*/*v*) [22,33,41]. The plates were visualized by UV illumination at 254 nm, the putative camptothecin spots with the same blue color and relative mobility as the authentic specimen (Cat. 7689-0 3-4) were considered, and their intensities were determined by Image J software package relative to the authentic example. The putative spots of silica containing camptothecin were scraped off, dissolved in methanol, and camptothecin was extracted as described by [22]. The purity and concentration of camptothecin were determined by HPLC (YOUNG In, Chromass, 9110+ Quaternary Pump, Korea) using a C18 reverse phase column (Eclipse Plus C18 4.6 mm × 150 mm, 3.5 μm, Cat. #959963-902) with isocratic mobile phase methanol/water (60:40 *v*/*v*) at a flow rate 1.0 mL/min for 20 min, scanned by photodiode array detector (DAD). The chemical identity and concentration of the putative camptothecin were confirmed from retention time and peak area of authentic example at λ_360_ nm [22].

### 2.3. UV-Vis, FT-IR, and LC-MS Analyses

The putative spots of camptothecin, were scraped from the silica plate, dissolved in methanol, and scanned by UV-vis spectrophotometer (RIGOL, Ultra-3000 Series) at λ_300–400_ nm. The concentration of the putative camptothecin was determined and compared to the authentic concentration of camptothecin, using methanol as blank baseline.

The FT-IR spectra of camptothecin were analyzed with a Bruker FT-IR Spectrometer in a range of 400–4000 cm^−1^ with KBr pellets.

The chemical structure of the extracted camptothecin was resolved from the ^1^H and ^13^C NMR analysis by JEOL (ECA-500II) 500 MHz NMR. The sample was dissolved in CDCl_3_, and the chemical shifts and coupling constants were expressed as parts per million (δ-scale) and hertz (Hz), respectively.

The chemical identity of putative camptothecin was analyzed by liquid chromatography tandem mass spectrometry (LC-MS/MS), with a Thermo Scientific LCQ Deca mass spectrometer and Hypersil Gold aQ (C18 column), with an electrospray source in positive-ion mode. The mobile phases A (0.1% formic acid), and B (acetonitrile in 0.1% formic acid) were used [22,33,48]. The gradient elution system was 2–98% mobile phase B over 30 min at a flow rate of 0.2 mL/min for 40 min. The chemical identity of the resolved signals was determined regarding their mass spectral fragmentation pattern and retention times by NIST mass spectral library.

### 2.4. Molecular Identification of the Recovered Endozoic Fungi

The recovered fungal endophytes from the sponges were morphologically identified regarding their micro-morphological and macromorphological features as adopted by the universal fungal identification keys [45,46,47,49]. The identity of the potent camptothecin producing fungi was molecularly confirmed from the sequence of their internal transcribed spacers (ITS) [40,50,51,52,53]. Genomic DNA (gDNA) from the potent fungal isolates was extracted by cetyl-trimethyl-ammonium bromide (CTAB) reagent [40,52]. The purity and concentration of the extracted gDNA was checked and determined by 1.5% agarose gel. The fungal gDNA was used as a template for PCR with the primer set; ITS5 5′-TCCTCCGCTTATTGATATGC-3′, ITS4 5′-GAAGTAAAAGTCGTAA-CAAGG-3′. The PCR reaction contains 10 μL of 2 × PCR master mixture (i-Taq™, Cat. No. 25027), 1 μL of gDNA, 1 μL of primers (10 pmol) and completed to 20 μL total volume. The PCR was programed at initial denaturation 94 °C for 2 min, followed by 35 cycles at denaturation 94 °C for 30 s, annealing 55 °C for 20 s, extension 72 °C for 40 s, and final extension 72 °C for 2 min. The PCR products were analyzed by 2% agarose gel, sequenced by an Applied Biosystems Sequencer, HiSQV Bases. The sequences were non-redundantly BLAST searched on NCBI, imported into MEGA 7.0 software and aligned by Clustal W muscle algorithm [54] and the phylogenetic relatedness was created by the neighbor-joining method [54,55].

### 2.5. Bioprocess Optimization of Camptothecin Production by the Potent Fungal Isolate with Plackett-Burman and Faced Central Composite Designs (FCCD)

Various physicochemical parameters, acid whey, malt extract, potato starch, methyl jasmonate, tryptamine, peptone, dextrin, tryptone, glucose, salicylic acid, tryptophan, serine, cysteine, pyruvate, phenylalanine, and glutamate, were optimized by Plackett-Burman design to maximize the yield of camptothecin from the tested fungal isolate [22,33,56]. The sixteen parameters were screened by two variables of Plackett-Burman design, each represented by high (+) and low (−) levels, according to the first order reaction:

*Y* = *β*0 + Σ*βiXi*

*Y* is the predicted yield of camptothecin, *Xi* is an independent variable, *βi* is the linear coefficient, and *β*0 is the model intercept. Triplicates for each run were conducted, and the average camptothecin yield was used as the main response. The highest significant independent variables controlling camptothecin productivity by the selected fungal isolate were optimized by Faced Central Composite design (FCCD) to determine the individual interactions of the tested variables [57,58]. With the FCCD, each variable was represented by three levels, low (−1), medium (0), high (+1), and the center point. The regression model showing the linear quadratic and interaction coefficients follows the second-ordered polynomial equation:

*Y* = *β*0 + Σ*i βiXi* + Σ*ii βiiXi* + Σ*ij βijXj*

where *Y* is the predicted response, *β*0 is the regression coefficient, *βi* is the linear coefficient, *βii* is the quadratic coefficient, and *Xi* is the coded level of independent variable.

### 2.6. Effect of Fungal Growth Inhibitors/Elicitors on Camptothecin Production by the Potent Fungal Isolate

The influence of different fungal growth inhibitors on triggering the camptothecin productivity of the selected fungal isolate was evaluated [22,33,42,59,60]. The fungal cultures were grown in the optimal media for 5 days at 30 °C, amended with different concentrations of fluconazole, griseofulvin, lamifen, methyl-jasmonate and NaCl at 1.0, 5.0, and 10.0 mM final concentration, then the cultures were further incubated for 15 days at standard conditions. Camptothecin was extracted and quantified by TLC and HPLC and described above [22].

### 2.7. Stability of Camptothecin Productivity during Fungal Storage, and Effect of Sponge Extracts on Restoring Biosynthetic Machinery

The biosynthetic stability of camptothecin productivity by *Penicillium chrysogenum* in response to storage was evaluated. The axenic fungal isolate “first isolated” has been stored as slope culture at 4 °C, and the camptothecin productivity was evaluated monthly for 10 months by growing the fungus on the optimized medium, followed by extraction and quantification of camptothecin by TLC and HPLC [22,33].

The influence of different organic extracts of the marine sponge. i.e., chloroform, methanol, ethyl-acetate and methylene chloride, on the productivity of camptothecin from the tested fungus was assessed [22,33]. Five grams of fresh sponge materials were grinded in 50 mL of each solvent, and kept at 4 °C for 12 h. The extracts were filtered, centrifuged at 5000 rpm, and concentrated by evaporation till 10 mL. Different volumes (0.5, 2 and 4 mL) of each extract were amended for the 5 days old culture, and incubation continued for 14 days under standard conditions. The same solvent extracts of the sponge in fungus-free media were used as negative control, along with normal control of fungal cultures without sponge extracts. After incubation of the culture under standard conditions, camptothecin was extracted and quantified as described above.

### 2.8. Antiproliferative Activity of the Purified Camptothecin from Penicillium Chrysogenum

The antiproliferative activity of the extracted camptothecin CPT against human Larynx carcinoma (HEP-2) and colon carcinoma (HCT-116) cell lines was determined by 3-(4,5-dimethylthiazol-2-yl)-2,5-diphenyl tetrazolium bromide (MTT) assay [61]. The 96 microtiter plate was seeded with 10^3^ cells/well, and incubated for 12 h at 37 °C, then amended with different concentrations of camptothecin, and the plates were re-incubated for 48 h. The MTT reagent (25 μL) was added, and the developed purple color of formazan complex was measured at λ_570_ nm after 2 h. The IC_50_ value was expressed by the amount of camptothecin suppressing the growth of 50% of the initial number of cells, normalizing to positive controls.

### 2.9. Internal Transcriped Spaer Fungal Deposition

The ITS sequence of *Penicillium chrysogenum* EFBL “an endozoic of *Cliona* sp.” was deposited on GenBank with accession # OL597937.1 (https://www.ncbi.nlm.nih.gov/nuccore/2154050751) (accessed on 22 March 2022).

### 2.10. Statistical Analysis

The experiments were conducted in biological triplicates and the results were expressed by means ± STD. The significance and F-test were calculated using one-way ANOVA with Fisher’s Least Significant Difference post hoc test.

## 3. Results

### 3.1. Isolation, and Screening for Camptothecin from Marine Sponges-Derived Fungi

**Figure 1:**
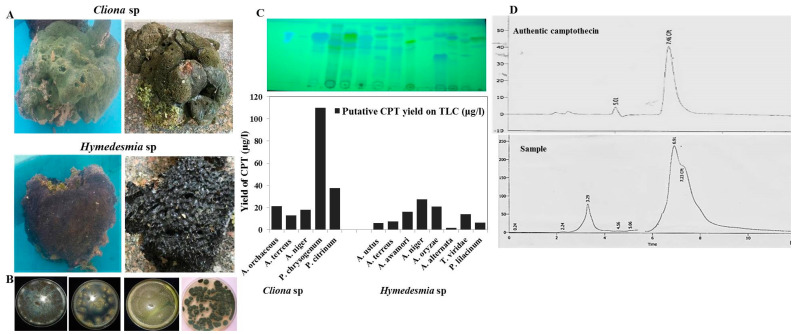
Screening for camptothecin producing fungal endophytes inhabiting marine sponges *Cliona* sp. and *Hymedesmia* sp. (**A**), General view of *Cliona* sp. and *Hymedesmia* sp. collected from the Red Sea, 20 km away from Sharm El sheikh, Egypt. (**B**), The collected fungal isolates from both sponges were grown on PDB for 10 days at 30 °C, then camptothecin was extracted by methylene chloride, checked by TLC, and visualized by UV at wavelength 254 nm comparing to authentic one. (**C**), TLC chromatogram of methylene chloride extracts from the fungal isolates (Upper panel), and the putative concentration of camptothecin calculated by the Image J software package, normalized to known concentration of authentic camptothecin (Lower panel). (**D**), HPLC chromatogram of extracted camptothecin and authentic one.

Two marine sponges, *Cliona* sp. and *Hymedesmia* sp., were isolated from the Red Sea at a depth of 10 m, and their fungal flora were isolated on PDA, Czapek’s-Dox and malt extract media. Thirteen fungal isolates were recovered from the marine sponges; five isolates were from *Cliona* sp. and eight were isolates from *Hymedesmia* sp. (Table 1). These fungal isolates were morphologically identified based on their macroscopical and microscopical features according to the universal keys of fungal identification. These fungal flora belonged to two genera, *Aspergillus* and *Penicillium*, at 70% and 25%, respectively. The camptothecin productivity from the fungi isolates derived from the sponges was assessed by growing on PDB medium, incubated for 10 days under standard conditions, then camptothecin was extracted and quantified by TLC and HPLC (Figure 1). From the screening paradigm, and the fungal isolates derived from *Cliona* sp, *Penicillium chrysogenum* was the highest camptothecin producer (110 µg/L), followed *P. citrinum* (38 µg/L), while the remaining fungal isolates exhibited a mild yield of camptothecin (0–10 µg/L). Practically, the productivity of the same species of the genus of *Aspergillus* derived from the different types of marine sponge was complete, ensuring the role of the fungal–animal interaction, and biological and physiological difference in manipulating the expression of camptothecin biosynthetic machinery of the potent fungus. Thus, the biological identity of marine sponge could make a significant contribution not only to the identity of their endogenous associated fungal flora, but also to the pattern of metabolic identity of the derived fungal isolates.

### 3.2. Molecular Identification of Potent Camptothecin Producing Fungi

**Figure 2:**
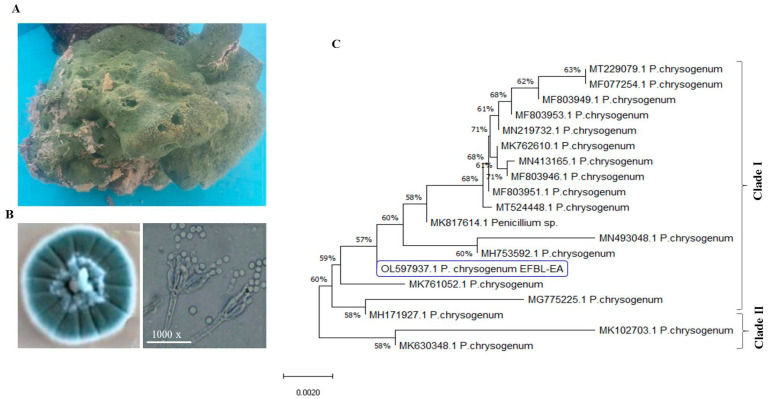
Identification of the potent camptothecin producing fungal isolate from *Cliona* sp. (**A**) Macromorphological features of the potent camptothecin producing isolate. (**B**) Microscopical features of the conidial heads of the fungal isolate at 1000× magnification. (**C**) Molecular phylogenetic analysis of the ITS sequence of the target isolate by Maximum Likelihood method.

The most potent camptothecin producing fungal isolate, *Penicillium chrysogenum* derived from *Cliona* sp. was further identified based on the sequence of their ITS regions. For the morphological description, the fungal isolate was grown on PDA and Malt Extract media for 10 days at 30 °C, and the macroscopical and microscopical features were observed daily. The fungal matter grew rapidly on PDA, with blue-brown, deep blue conidial pigmentation and mycelia color, with yellow edges of the colonies and absence of soluble exudates, and with a brown reverse on the colonies (Figure 2). The fungus has a 2–4 divergent Penicilli, with bi-verticillate branching pattern, green conidia, globose, sub-gobose conidial heads and smoothed condidial cell walls. These morphological features are closely matched with the descriptions of *P. chrysogenum* [46,49,56,62,63]. The morphological identity of the isolate *P. chrysogenum* has been confirmed from the molecular sequence of the ITS region. The gDNA of the fungus was used as the template for PCR, and the PCR amplicon was around 650 bp (Figure 2). The PCR amplicon was purified and sequenced, and BLAST searched non-redundantly on the GenBank. The target sequence displayed a 99.9% similarity with the deposited sequence of *P. chrysogenum*, with zero E value. The ITS sequence of the fungus was deposited in the GenBank with accession # OL597937.1. From the BLAST and phylogenic analysis, two cluster of *Penicillium* sp. arose, Cluster I and Cluster II (Figure 2). The ITS sequence of *P. chrysogenum* EFBL displayed a 99.9% similarity with the *P. chrysogenum* isolates of accession numbers MK761052.1, MG775225.1, MH171927.1, MH753592.1, MN4903048.1, MK817614.1, MT524448.1, MF803946.1, MN413165.1, MN219732.1, MF803953.1, MF803949.1 and MF077254.1 and MT229079.1, while the ITS sequence of current fungus displayed a 99.0% similarity with *P. chrysogenum* MK102703.1 and MK630348.1. Taken together, from the morphological and molecular analyses of the ITS sequence, the potent camptothecin producing isolate derived from *Cliona* sp. has been confirmed as *P. chrysogenum*.

### 3.3. Chromatographic and Spectroscopic Analyses, and Antiproliferative Activity of Extracted Camptothecin

**Figure 3:**
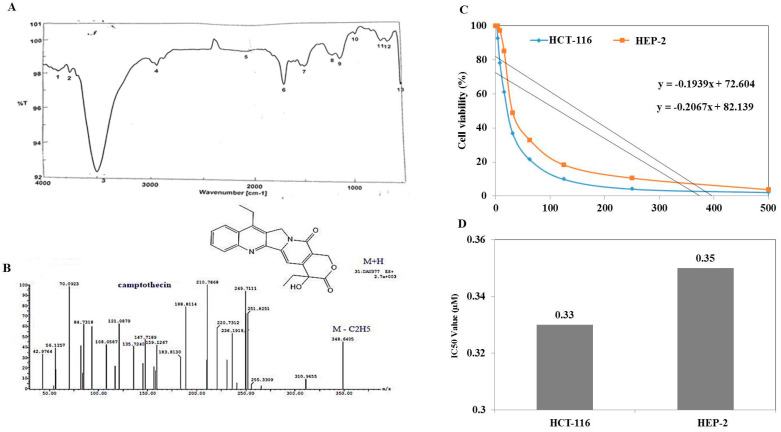
Chemical analysis and anticancer activity of *P. chrysogenum* extracted camptothecin. After growing *P. chrysogenum* under standard conditions, camptothecin was extracted and checked by TLC, putative spots scraped off, and camptothecin extracted and chemically analyzed. (**A**) FTIR chromatogram of extracted camptothecin. (**B**) LC-MS/MS analyses of extracted camptothecin. The antiproliferative activity of extracted camptothecin against human larynx carcinoma (HEP-2) and colon carcinoma cell lines as revealed from the viability plot (**C**) and IC_50_ values (**D**).

The chemical identity of the putative camptothecin from *P. chrysogenum* was confirmed from the TLC, HPLC, and LC-MS analyses, comparing to the authentic sample. Camptothecin was extracted, fractionated by TLC, and the silica gel spots containing camptothecin were scraped off and dissolved in methanol for chemical analysis. From the HPLC chromatogram, the putative sample of camptothecin gave the same retention time (7.42 min) as the authentic one (Figure 3), ensuring its chemical proximity as camptothecin. From the UV-absorption spectra (Figure 3), the putative camptothecin sample from *P. chrysogenum* displayed the maximum absorption peak at absorbance 290 nm, identical to the standard absorption spectrum of camptothecin. The chemical identity of *P. chrysogenum* camptothecin was verified from the ^1^HNMR, displaying the same resolved signals as the authentic one, distributed between 1.0 and 8.0 ppm, with three proton signals resolved at 1.0–2.5 ppm corresponding to methyl, acetate and acetylene groups. From the FTIR spectra, the extracted *P. chrysogenum* camptothecin had peaks at 3406.6 and 3393.3 cm^−1^, assigned for the hydroxyl (OH) and amide group stretches, respectively. Peaks of 2923.56, 1729.83 and 1604.5 cm^−1^ were assigned to the aliphatic CH, ester groups and aromatic rings stretch, respectively. The COO stretching frequency peaked at 1268.9 cm^−1^, while peak at 1029.8 cm^−1^ was assigned for the aromatic C and H blends (Figure 3).

**Figure 4:**
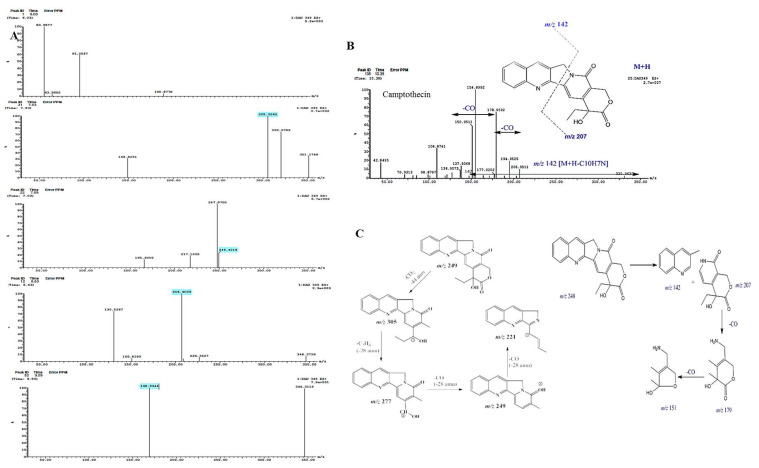
Chemical validation of the extracted camptothecin from *P. chrysogenum*. The putative spots of camptothecin were scraped-off from the TLC, and its purity was checked by HPLC. (**A**), LC-MS/MS fragmentation pattern of putative camptothecin from *P. chrysogenum*. (**B**), LC MS/MS spectra of the putative purified camptothecin with the onset chemical structure of camptothecin. (**C**), Scheme of molecular fragmentation pattern of camptothecin as revealed by the LC-MS/MS spectra.

The chemical structure of camptothecin has been confirmed by UPLC-ESI-MS/MS analysis positive mode. From the LC-MS/MS, the camptothecin of *P. chrysogenum* gave the same molecular mass to charge ratio (348.2 *m/z*), in addition to the same molecular fragmentation pattern as standard camptothecin (Figure 4) and camptothecin from *Camptotheca acuminata* (Wall et al., 1966). The parent camptothecin (348.2 *m/z*) was further fragmented by a second LC-MS applying collision energy of 35 electron Volts (eV); fragments of 94.7, 128.9, 142.9, 165.1, 200.7, 216.14, and 228.9 *m/z* were recovered, with the same fragmentation pattern as the authentic example. From the profile of the first mass spectra, a peak at retention time 10.38 min with a molecular ion peak at *m/z* 349 [M + H]+ corresponding to the molecular formula C_20_H_16_N_2_O_4_ in addition to other diagnostic peaks of camptothecin alkaloid. Ethyl camptothecin at retention time 24.96 min showed a molecular ion peak at *m/z* 377 [M + H]+ followed by loss of ethyl group and camptothecin fragmentation. The peaks at retention times 10.38, 10.97, 10.98 and 12.49 min exhibited a protonated molecular ion peak [M + H]+ at *m/z* 349, and ESI-MS/MS fragment ion at *m/z* 207 is produced by the cleavage of C_10_H_7_N. The fragment ions at *m/z* 179 and 151 show losses of two carbonyl group moieties, ensuring the chemical identity of the target compound as camptothecin. Thus, from the TLC, HPLC, FT-IR, LC–MS/MS, and UV-absorption spectra, the putative camptothecin from *P. chrysogenum* derived from *Cliona* sp. has been chemically authenticated as camptothecin.

The antiproliferative activity of extracted camptothecin from *P. chrysogenum* was evaluated towards different cell lines, human larynx carcinoma (HEP-2) and colon carcinoma (HCT-116 (Figure 3) by MTT assay. The IC_50_ values of extracted camptothecin of *P. chrysogenum* were 0.33–0.35 µM towards the tested cell lines.

### 3.4. Bioprocess Optimization of Camptothecin Production by P. chrysogenum with Plackett-Burman Design

**Figure 5:**
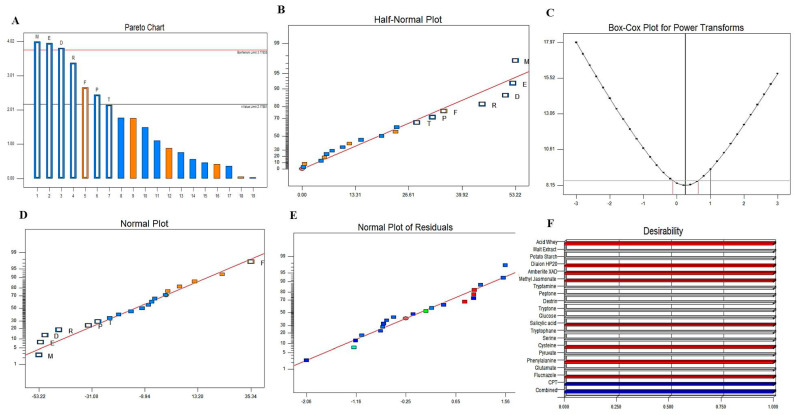
The main effects of different variables on camptothecin production according to the Plackett-Burman experimental design. The normal probability plots of the variables for camptothecin production by *P. chrysogenum* as determined by the first order polynomial equation. (**A**) Pareto chart illustrates the order of significance of each variable. (**B**) Plot of correlation between predicted and actual camptothecin yield of *P. chrysogenum*. (**C**), Box-Cox power transform. (**D**) Plot of standardized effect with normal probability. Plot of standardized effect with normal residuals (**E**) and desirability (**F**).

Among the tested isolates, *P. chrysogenum* was selected for further nutritional optimization to maximize its yield of camptothecin. The chemical identity of medium components and their interactions have a pivotal role in the regulation of the biosynthetic machineries of secondary metabolites by fungi. Consequently, the medium components were nutritionally optimized by response surface methodology with Plackett-Burman’s design as “first order model equation” to maximize the yield of camptothecin. Sixteen runs were conducted to assess the effect of the different variables on camptothecin productivity for *P. chrysogenum* (Table 2). These variables include different carbon and nitrogen precursors of camptothecin in addition to growth modulators and elicitors. The significance of the tested independent variables influencing camptothecin productivity by *P. chrysogenum*, with the predicted and corresponding actual responses, was summarized in the Plackett-Burman design matrix (Table 3). The actual and predicted yield of camptothecin by *P. chrysogenum* noticeably fluctuated from 50.3–203.5 µg/L, confirming the significance of the tested variables on camptothecin biosynthesis, and the efficiency of the Plackett-Burman design, the values of the coefficient of determination (R^2^ = 0.98) indicating the goodness-of-fit measure for the linear regression models (Table 4). The variability in the response of camptothecin production was attributed to the selected independent variables but the remaining variations were (0.01%). The values of the adjusted determination coefficient (Adj. R^2^ = 0.92), F-value (16.524) and F-value (0.02 < 0.05) reveal the significance of this model. The analysis of variance (ANOVA) of the experimental design results was calculated and the coefficients, t Stat, *p*-value, and confidence levels were also recorded (Table 4). The main effects and the normal probability of the tested variables were measured and plotted (Figure 5), indicating that there are five different independent factors, Phenylalanine (X15), Pyruvate (X14), Methyl Jasmonate (X4), Salicylic acid (X10) and cysteine (X13), that positively influence camptothecin yield, while the other independent factors have a negative effect. The highest camptothecin yield (199 μg/L) was recorded at run 14, while the lowest value (52 μg/L) was observed in run 7. The significance of the variables affecting camptothecin productivity by *P. chrysogenum* is displayed in the Pareto Chart, as well as the probability plot of independent variables, actual and predicted yield of camptothecin (Figure 5). The significance of each variable was assessed from the p-value and student’s t-test as reported in Table 4. The arrangement of the points of residuals around the diagonal line shows the independent normal distribution of the variables, suggesting the perfect fitting of predicted and actual camptothecin yield. From the ANOVA analysis, the constructed model was highly significant, as shown from the values of Fisher’s F-test 3.3 and probability p-value 0.0335. The first order polynomial equation of camptothecin production by *P. chrysogenum* regarding the significant independent variables was derived from the following equation:

Final equation in terms of actual factors affecting camptothecin productivity = 360.54833 − 16.9133 ∗ Phenylalanine + 17.53333 ∗ Pyruvate + 88.35 ∗ Methyl Jasmonate − 26.61 ∗ Salicylic acid − 5.42333 ∗ Cysteine − 11.23 ∗ Phenylalanine − 9.53333 ∗ Fluconazole.

The highest actual (199 µg/L) and predicted yield (203 µg/L) of camptothecin by *P. chrysogenum* was detected at run # 14, with the residual value—4.8. At run # 14, the maximum yield of camptothecin was detected at higher concentration variables of phenylalanine (5 g/L), pyruvate (20 g/L), methyl jasmonate (20 g/L), salicylic acid (4 g/L), cysteine (10 g/L), phenylalanine (8 g/L) on the 15th incubation day at 30 °C, with lower doses of other remaining variables (Table 4). Thus, upon using Plackett-Burman design, the yield of camptothecin by *P. chrysogenum* was increased by about 1.8 fold (200 µg/L) compared to control “non-optimized” fungal cultures (110.6 µg/L). A similar optimization protocol has been developed to optimize the camptothecin productivity by *A. terreus* and *A. flavus* (24), ensuring the successfulness of the optimization process by the response surface methodology of the cultural variables compared to classical one-one factor optimization.

### 3.5. Effect of Fungal Growth Inhibitors on Camptothecin Yield by P. chrysogenum

**Figure 6:**
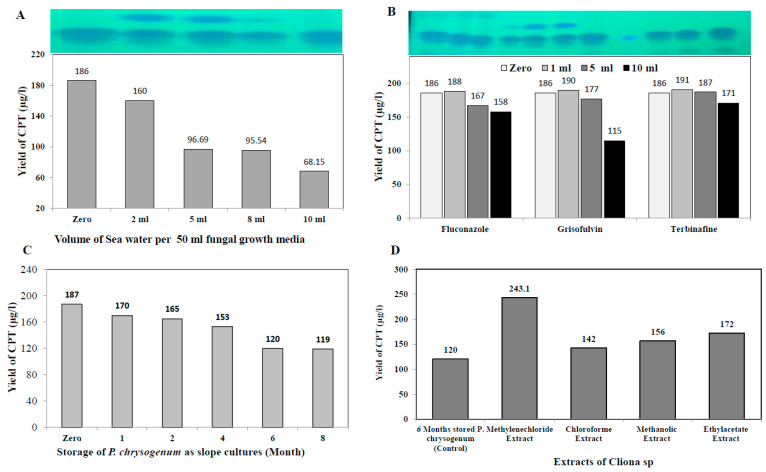
Effect of sea water concentration and growth inhibitors on camptothecin productivity for *P. chrysogenum*. The yield of camptothecin for *P. chrysogenum* in response to different sea water concentration (**A**), and growth inhibitors Grisofulvin, terbinafine (**B**). (**C**) The productivity of camptothecin for *P. chrysogenum* in response to fungal storage periods as slope culture. (**D**) The yield of camptothecin for *P. chrysogenum* in response to amendment with different extracts of *Cliona* sp.

The effect of incorporation of fungal growth inhibitors griseofulvin, terbinafine, and fluconazole (1, 5, and 10 mM final conc.) on camptothecin yield by *P. chrysogenum* was assessed. The fungal isolate was grown on the optimized media, the different concentrations of each compound were added, and the cultures were continue incubated for 14 days; camptothecin was extracted and quantified by HPLC. From the obtained results (Figure 6), there is no obvious inducing effect by inhibitors on the biosynthetic machinery of camptothecin for *P. chrysogenum*. The lack of inducing effect of these inhibitors on the biosynthetic machinery of camptothecin ensures the independence of camptothecin biosynthetic systems for this fungus on the external stimuli. Since the fungus has been isolated from the marine sponge, the effect of salinity might play a role on inducing the expression system for the biosynthesis of camptothecin. To validate this hypothesis, different salt concentrations were amended to the cultures, and the yield of camptothecin was evaluated as described above. From the results (Figure 6), an obvious decrease in the camptothecin yield by *P. chrysogenum* was recorded upon addition of NaCl. The highest yield of camptothecin was determined in the absence of NaCl, while the yield of camptothecin decreased by about 50% upon addition of 100 mM NaCl to the culture media of *P. chrysogenum*.

### 3.6. Biosynthetic Stability of Camptothecin by P. chrysogenum in Response to Storage, and Effect of Sponge Extracts on Restoring Its Biosynthetic Machinery

The biosynthetic stability of camptothecin productivity by *Penicillium chrysogenum* in response to storage was evaluated. The axenic first fungal isolate, maintained as slope culture on PDA at 4 °C, was grown on the optimized media, and their camptothecin productivity over a 10 month interval was determined by standard conditions. From the results (Figure 6), it can be seen that the camptothecin productivity slightly decreased with storage of the fungus as slope culture at 4 °C. After 8 months of storage at 4 °C, the residual camptothecin productivity for *P. chrysogenum* was reduced to 119 μg/L, i.e., about a 40% loss, compared to zero culture (188 μg/L). A noticeable decrease in camptothecin productivity for *P. chrysogenum* was observed with fungal storage, consistent with the overall physiological features of secondary metabolites’ attenuation by fungi [33,40,41,42,56,62]. Remarkably, a visual fading to the mycelial pigmentation of *P. chrysogenum* was observed with fungal storage, strongly matched with the sequential loss of camptothecin productivity, ensuring the metabolic and genomic correlation of both biosynthetic pathways of camptothecin and melanin pigments [64].

**Figure 7:**
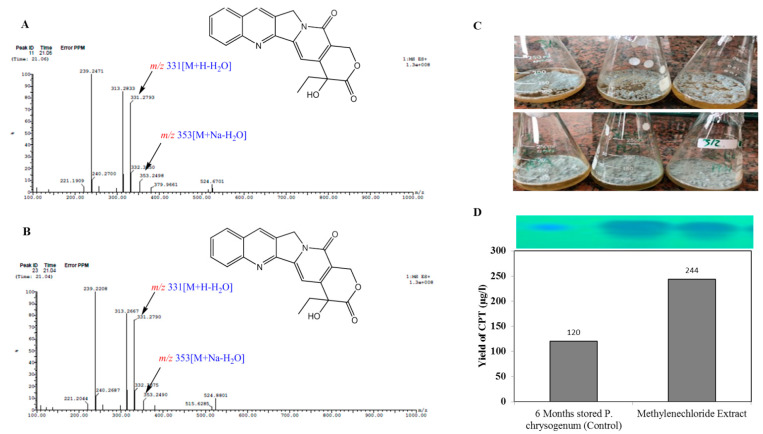
Restoring the biosynthetic potency of camptothecin of the 6-month stored *P. chrysogenum* upon addition of methylene chloride of *Cliona* sp. The 6-month stored *P. chrysogenum* was grown on the optimized medium, and methylene chloride extracts of *Cliona* sp. were amended to the culture after 5 days of incubation under standard conditions, then camptothecin was extracted and checked by LC-MS/MS. The LC-MS/MS pattern of extracted camptothecin from the control culture of *P. chrysogenum* (6-months) (**A**) and culture amended with methylene chloride extracts of *Cliona* sp. (**B**). (**C**) Morphological features showing conidial pigmentation of control culture (upper panel) and amended with methylene chloride extracts of *Cliona* sp. (lower panel). (**D**) Yield of camptothecin from the cultures of *P. chrysogenum* as determined by HPLC.

The influence of different organic extracts, chloroform, methanol, ethyl-acetate and methylene chloride, for the marine sponge “*Cliona* sp.” on restoring the productivity of camptothecin by *P. chrysogenum* was assessed. The extracts of *Cliona* sp. were amended to 6 month stored *P. chrysogenum*, and the fungal productivity of camptothecin was assessed by TLC, HPLC and LC-MS analyses. From the LC-MS profile (Figure 7), methylene chloride extracts of *Cliona* sp. have the maximum potency in restoring the camptothecin biosynthetic machinery of *P. chrysogenum* (Figure 6), with a lack of inducing effect in the other tested extracts. With methylene chloride extract, the yield of camptothecin was increased to 244 μg/L, i.e., by about 2.1 fold, compared to the 6 month stored *P. chrysogenum* culture (120 μg/L). From the profile of LC-MS, several metabolic intermediates were induced upon addition of sponge extracts, ensuring the dependence of the expression of a plethora of metabolites on the signals derived from the sponge, and these signals are mainly extracted from methylene chloride. Thus, it could be deduced that the expression of the camptothecin biosynthetic machinery of *P. chrysogenum* could be partially dependent on the chemical signals derived from the sponge, or from the associated microbial flora of the sponge.

## 4. Discussion

Camptothecin is one of the most powerful alkaloids for cancer therapy due to its unique affinity for binding with DNA topoisomerase I [9,65], blocking its various biological processes: DNA replication, RNA transcription and chromatin assembly. Camptothecin derivatives are one of the most commonly prescribed anticancer drugs, comparable to Taxol and vincristine, mainly extracted from the plant *Camptotheca acuminata*, inhabiting China and India [8]. However, the tiny yield of camptothecin from natural plant sources, difficulties in extraction, the vulnerability of the yield of this plant to environmental and ecological conditions, and the massive harvesting of the plant causing destruction to the ecological balance are all major challenges [1,66]. Nevertheless, with the emergence of endophytic fungi as an eminent producer of camptothecin, a new promising platform has been raised for the fungal fast growth, feasibility of mass production, independence from environmental conditions and feasibility of metabolic engineering [32,33,64,67]. However, the tiny yield and loss of the camptothecin biosynthetic potency of fungi during storage and subculture are the main challenges that halt the further industrial application of fungi [22,33,41,59,64,68]. Thus, searching for novel camptothecin producing fungal isolates, with metabolic/biosynthetic stability and sustainability for camptothecin biosynthesis, is the main objective of this work. Recently, marine fungi have been reported as an untapped repertoire for novel bioactive secondary metabolites, with diverse biological and pharmaceutical activities [34]. However, the biological and metabolic identities of marine fungi have received less attention and less exploration, compared to their terrestrial counterparts [35]. Thus, marine fungi could be a potential candidate for discoveries of novel compounds with unique chemical skeletons and scaffolds that can be modified to produce novel bioactivity and pharmaceutical activity. The rationality of producing a wide range of metabolites from marine fungi could be attributed to the diverse ecological, physical and biological factors that could be epigenetic regulators of the biosynthetic gene clusters of secondary metabolites. Sponges are one of the most recognized reservoirs of marine fungi, and about 40–60% of the sponge biomass mainly consists of associated microorganisms, along with sponge feeding [69]. Several marine fungi associated with the marine sponge were isolated and partially characterized [70,71]. Several types of Mediterranean marine sponges *Ircinia variabilis, Suberites zeteki and Mycale armata* were studied and their fungal associations were described [34,37,72]. Thus, isolating novel fungal isolates producing camptothecin, while evaluating their potential metabolic stability for camptothecin biosynthesis, is the objective of this study.

The marine sponges *Cliona* sp. and *Hymedesmia* sp. were collected from the Red Sea at a depth 10 m, and their fungal flora were isolated. Five isolates associated with *Cliona* sp. and eight isolates derived from *Hymedesmia* sp. were isolated. From the screening profile, *Penicillium chrysogenum* associated with *Cliona* sp. was the highest camptothecin producer, followed by *P. citrinum*. Practically, the yield of camptothecin of *P. chrysogenum* associated with *Cliona* sp. is much higher than that of the closely related isolate *P. lilacinum* associated *Hymedesmia* sp., ensuring the role of fungal–animal interaction and biological and physiological differences in manipulating the expression of the camptothecin encoding gene cluster. This is the first report describing the fungal flora of the marine sponges *Cliona* sp. and *Hymedesmia* sp., ensuring the metabolic uniqueness of their associated fungal flora. The morphological identity of *P. chrysogenum* EFBL, derived from *Cliona* sp., has been molecularly confirmed based on the ITS sequence, and deposited on GenBank with accession # OL597937.1. From the literature, this is the first report describing the metabolic potency of the marine fungal derived fungus *P. chrysogenum* as camptothecin producer that could produce novel biosynthetic machinery for sustainable camptothecin production. The yield of camptothecin for *P. chrysogenum* (110 μg/L), derived from *Cliona* sp., is higher than that of *Aspergillus terreus* (90 μg/L) and *A. flavus* (75 μg/L) endophytes of *Ficus elastica*, and *A. fumigatus* (35 μg/L) endophytes of *Delonix regia* [22,73]. The yield of camptothecin for *P. chrysogenum* is higher than that reported for *A. flavus* (51.7 µg/L) and *A. flavus* (37.2 µg/L), an endophyte of *Astragalus fruticosus.* The higher yield of endozoic associated *P. chrysogenum* from the sponge *Cliona* sp. could be ascribed to the diverse chemical signals due to fungal–animal interactions, or the identity of microbiome interactions and other related chemical signals that trigger the expression of camptothecin encoding genes. Similar results confirm the presence of the camptothecin biosynthetic machinery of *A. flavus*, an endophyte of *Ficus elastica*, using the same screening paradigm [22,59,62,73].

The chemical identity of the putative camptothecin from *P. chrysogenum* has been confirmed. From the HPLC and UV-absorption spectra, the camptothecin sample had the same retention time and maximum absorption peak as the authentic substance. From the LC-MS/MS, camptothecin of *P. chrysogenum* gave the same molecular mass to charge ratio (348.2 m/z), as well as the same molecular fragmentation pattern as the authentic. Similar instances have been reported, confirming the chemical structure of camptothecin from various plant and fungal sources using the same chromatographic and spectroscopic approaches [19,20,21,22,24,74]. The anticancer activity of *P. chrysogenum* camptothecin was evaluated against different cell lines of human larynx carcinoma (HEP-2) and colon carcinoma (HCT-116) with IC50 values 0.33–0.35µM, higher than A. flavus camptothecin against HEPG-2 (IC50, 0.9 mM), MCF7 (IC50, 1.2 µM), and HCT29 (IC50, 1.35 µM) [22]. Consistently, the IC50 values of *P. chrysogenum* camptothecin were similar to A. terreus camptothecin towards MCF7 (0.18 mM), LS174 T (0.29 µM), HCT29 (0.43 µM) and HEPG-2 (0.73 µM) [75,76,77].

The yield of camptothecin by *P. chrysogenum* has been maximized by nutritional optimization of the medium components to assess their interactions in regulating the biosynthetic machinery of secondary metabolites by fungi [40,52,59,64,78,79]. The variables, including various proportions of carbon and nitrogen, as well as growth modulators and elicitors, were optimized by Plackett-Burman design to maximize the yield of camptothecin. From the probability of the tested variables, independent factors, especially phenylalanine, pyruvate, methyl Jasmonate, salicylic acid and cysteine, have a positive influence on camptothecin yield. The maximum actual and predicted yield (203 µg/L) of camptothecin for *P. chrysogenum* was assessed at run # 14, with the residual value 4.8. The yield of camptothecin for *P. chrysogenum* increased by about 1.8 fold comparing to non-optimized fungal cultures. A similar optimization protocol has been developed to optimize the camptothecin productivity for *A. terreus* and *A. flavus* [22,33,64,80], ensuring the success of the optimization process by the response surface methodology of the cultural variables compared to classical one-one factor optimization. Similar optimization protocols have been developed to maximize camptothecin productivity by *Fusarium solani*, *Nothapodytes nimmoniana* and *Trichoderma atroviride* [18,69,81]. Upon addition of methyl-jasmonate, the yield of camptothecin was significantly increased by about 1.6 fold compared to control, this being consistent with the results of camptothecin production by *Trichoderma atroviride* [82] and *A. terreus* and *A. flavus* (El-Sayed et al. 2021). Methyl-jasmonate is the most recognized elicitor, causing crosstalk with plasma membrane receptors, inducing arrays of defense responses including reactive oxygen and nitrogen oxygen species that subsequently induce the expression of secondary metabolites, encoding genes [20,33,83]. The effect of various growth inhibitors on camptothecin productivity for *P. chrysogenum* was assessed. There is no obvious positive effect of these inhibitors on the biosynthetic machinery of camptothecin by *P. chrysogenum*, ensuring their lack in the biosynthetic machinery, independently of this fungus, of the external stimuli. The biosynthetic stability of camptothecin productivity by *Penicillium chrysogenum* in response to storage has been evaluated. The camptothecin productivity slightly decreased during the storage of the fungus; after 8 months of storage at 4 °C, the camptothecin productivity for *P. chrysogenum* decreased by about 40% compared to the first culture. Unlike the rapid attenuation of camptothecin productivity by *A. terreus* and *A. flavus* [33,64,82,83], the camptothecin biosynthetic machinery for *P. chrysogenum* was slightly stable. The overall biosynthetic attenuation of the secondary metabolites by fungi have been frequently reported [21,24,30,33,64,75,82,83]. The influence of different organic extracts, chloroform, methanol, ethyl-acetate and methylene chloride, from the marine sponge “*Cliona* sp.” on restoring the productivity of camptothecin for *P. chrysogenum* was assessed. From the LC-MS analysis, it can be seen that methylene chloride extracts of *Cliona* sp. have the potency to restore the camptothecin biosynthetic machinery of *P. chrysogenum*, and the yield of camptothecin was increased to 244 μg/L. Several metabolic intermediates were induced upon addition of methylene chloride sponge extracts, ensuring the dependence of the expression of a plethora of metabolites on the signals derived from sponge, or from the sponge associated microbiome.

In conclusion, *P. chrysogenum*, endozoic of marine sponge *Cliona* sp., was a potent camptothecin producer. Remarkably, the biosynthetic machinery of camptothecin was relatively stable during fungal storage and subculturing. The slight attenuation of the fungal biosynthetic machinery has been completely restored with the addition of methylene chloride extract of sponge *Cliona* sp., ensuring the presence of specific signals derived from the sponge tissues, or signals from the associated microbiome of *Cliona* sp. Further studies are ongoing to uncover the molecular and metabolic identity of camptothecin and the biosynthetic machinery of *P. chrysogenum* using differential transcriptomic and proteomic approaches in order to stabilize their yield, paving the way for a novel platform for industrial camptothecin production.

## Figures and Tables

**Table 1:** Screening for CPT producing endophytes from marine sponges.

			CPT Yield on TLC (μg/L)
*Cliona* sp.	1	*A. orchaceous*	21.2
	2	*A. terreus*	12.8
	3	*A. niger*	18.2
	4	*P. chrysogenum*	110.1
	5	*P. citrinum*	37.8
*Hymedesmia* sp.	1	*A. ustus*	5.9
	2	*A. terreus*	7.6
	3	*A. awamori*	16.3
	4	*A. niger*	27.4
	5	*A. oryzae*	21.1
	6	*A. alternata*	1.7
	7	*T. viridae*	14.2
	8	*P. lilacinum*	6.6

**Table 2:** The coded and actual values for the tested variables affecting CPT production by *P. chrysogenum*.

Codes	Factors	Levels *	
		−1	1
**X1**	Acid Whey	2	5
**X2**	Malt Extract	1	3
**X3**	Potato starch	5	10
**X4**	Methyl jasmonate	0.1	0.5
**X5**	Tryptamine	1	2
**X6**	Peptone	2	1
**X7**	Dextrin	1	2
**X8**	Tryptone	5	10
**X9**	Glucose	5	10
**X10**	Salicylic acid	1	2
**X11**	Tryptophan	2	5
**X12**	Serine	1	4
**X13**	Cysteine	2	5
**X14**	Pyruvate	2	5
**X15**	Phenylalanine	2	4
**X16**	Glutamate	5	10

* The signs “−1” and “+1” refer to the minimum and maximum level of the tested parameters.

**Table 3:** Matrix of Plackett-Burman experimental design for camptothecin production by *P. chrysogenum*.

Run	X1	X2	X3	X4	X5	X6	X7	X8	X9	X10	X11	X12	X13	X14	X15	X16	CPT Yield (µg/L)	Predicted CPT yield (µg/L)	Residuals
**1**	−1	−1	1	1	−1	1	1	−1	−1	−1	−1	1	−1	1	−1	1	70.6	100.2	−29.6
**2**	−1	−1	−1	1	−1	1	−1	1	1	1	1	−1	−1	1	1	−1	80.2	40.8	39.4
**3**	−1	1	1	−1	1	1	−1	−1	−1	−1	1	−1	1	−1	1	1	93.6	120	−26.4
**4**	1	−1	−1	1	1	−1	1	1	−1	−1	−1	−1	1	−1	1	−1	92.7	126.9	−34.2
**5**	1	−1	1	1	1	1	−1	−1	1	1	−1	1	1	−1	−1	−1	80.3	90.0	−9.7
**6**	−1	1	−1	1	1	1	1	−1	−1	1	1	−1	1	1	−1	−1	81.2	98.0	−16.8
**7**	1	−1	1	1	−1	−1	−1	−1	1	−1	1	−1	1	1	1	1	52.1	87.9	−35.8
**8**	1	1	−1	1	1	−1	−1	−1	−1	1	−1	1	−1	1	1	1	82.2	79.0	3.2
**9**	1	−1	−1	−1	−1	1	−1	1	−1	1	1	1	1	−1	−1	1	96.1	96.4	−0.3
**10**	−1	1	−1	1	−1	1	1	1	1	−1	−1	1	1	−1	1	1	89.2	92.6	−3.4
**11**	1	1	1	1	−1	−1	1	1	−1	1	1	−1	−1	−1	−1	1	100.6	98.9	1.7
**12**	1	1	1	−1	−1	1	1	−1	1	1	−1	−1	−1	−1	1	−1	103.9	95.0	8.9
**13**	−1	1	1	−1	−1	−1	−1	1	−1	1	−1	1	1	1	1	−1	120.8	110.9	9.9
**14**	1	1	−1	−1	1	1	−1	1	1	−1	−1	−1	−1	1	−1	1	199.1	203.9	−4.8
**15**	−1	−1	−1	−1	1	−1	1	−1	1	1	1	1	−1	−1	1	1	102.2	98.7	3.5
**16**	1	−1	1	−1	1	1	1	1	−1	−1	1	1	−1	1	1	−1	181.2	128.3	52.9
**17**	−1	1	1	1	1	−1	−1	1	1	−1	1	1	−1	−1	−1	−1	180.4	160.2	20.2
**18**	−1	−1	1	−1	1	−1	1	1	1	1	−1	−1	1	1	−1	1	115.4	94.9	20.5
**19**	1	1	−1	−1	−1	−1	1	−1	1	−1	1	1	1	1	−1	−1	120.4	101.6	18.8
**20**	−1	−1	−1	−1	−1	−1	−1	−1	−1	−1	−1	−1	−1	−1	−1	−1	125.5	109.0	16.5

**Table 4:** Regression statistics and analysis of variance (ANOVA) for Placket-Burman design.

Source	Sum of Squares	df	Mean Square	F Value	*p*-Value Prob > F	
**Model**	66586.05	7	9512.29	10.85	0.0002	significant
**D-Phenylalanine**	12872.74	1	12872.74	14.68	0.0024	
**E-Pyruvate**	13833.8	1	13833.8	15.78	0.0019	
**F-Methyl Jasmonate**	6244.58	1	6244.58	7.12	0.0205	
**M-Salicylic acid**	14161.84	1	14161.84	16.15	0.0017	
**P-Cysteine**	5294.26	1	5294.26	6.04	0.0302	
**R-Phenylalanine**	10089.03	1	10089.03	11.51	0.0053	
**T-Fluconazole**	4089.8	1	4089.8	4.67	0.0517	
**Residual**	10520.32	12	876.69			
**Cor Total**	77106.37	19				
	**Coefficient**		**Standard** **Error**	**95% CI**		**VIF**
**Factor**	**Estimate**	**df**		**Low**	**High**	
**Intercept**	47.95	1	6.620776	33.52457	62.37543	
**D-Pyruvate**	−25.37	1	6.620776	−39.7954	−10.9446	1
**F-Methyl Jasmonate**	17.67	1	6.620776	3.244568	32.09543	1
**M-Salicylic acid**	−26.61	1	6.620776	−41.0354	−12.1846	1
**P-Cysteine**	−16.27	1	6.620776	−30.6954	−1.84457	1
**R-Phenylalanine**	−22.46	1	6.620776	−36.8854	−8.03457	1
**T-Fluconazole**	−14.3	1	6.620776	−28.7254	0.125432	1

Adeq Precision” measures the signal to noise ratio. A ratio greater than 4 is desirable. A ratio of 10.015 indicates an adequate signal. This model can be used to navigate the design space.

## Data Availability

All datasets generated for this study are included in the manuscript.

## Sample Availability

Samples of the compounds are available from the authors.
